# RETRACTION: Comparing the Traditional and Emerging Therapies for Enhancing Wound Healing in Diabetic Patients: A Pivotal Examination

**DOI:** 10.1111/iwj.70473

**Published:** 2025-04-02

## Abstract

**RETRACTION:** LiaoC. , ZhuM. , DingH. , LiY. , SunQ. , and LiX. , “Comparing the Traditional and Emerging Therapies for Enhancing Wound Healing in Diabetic Patients: A Pivotal Examination,” International Wound Journal 21, no. 3 (2024): e14488, 10.1111/iwj.14488.37984812 PMC10898383

The above article, published online on 20 November 2023, in Wiley Online Library (http://onlinelibrary.wiley.com/), has been retracted by agreement between the journal Editor in Chief, Professor Keith Harding; and John Wiley & Sons Ltd. Following an investigation by the publisher, all parties have concluded that this article was accepted solely on the basis of a compromised peer review process. The editors have therefore decided to retract the article. The authors did not respond to our notice regarding the retraction.

**RETRACTION:** C. Liao , M. Zhu , H. Ding , Y. Li , Q. Sun , and X. Li , “Comparing the Traditional and Emerging Therapies for Enhancing Wound Healing in Diabetic Patients: A Pivotal Examination,” International Wound Journal 21, no. 3 (2024): e14488, 10.1111/iwj.14488.37984812 PMC10898383

The above article, published online on 20 November 2023, in Wiley Online Library (http://onlinelibrary.wiley.com/), has been retracted by agreement between the journal Editor in Chief, Professor Keith Harding; and John Wiley & Sons Ltd. Following an investigation by the publisher, all parties have concluded that this article was accepted solely on the basis of a compromised peer review process. The editors have therefore decided to retract the article. The authors did not respond to our notice regarding the retraction.

